# Diversity and Functional Insights into Endophytic Fungi in Halophytes from West Ordos Desert Ecosystems

**DOI:** 10.3390/jof11010030

**Published:** 2025-01-04

**Authors:** Xingzhe Wang, Yan Zhang, Jingpeng Li, Yiteng Ding, Xiaodan Ma, Peng Zhang, Haijing Liu, Jie Wei, Yuying Bao

**Affiliations:** 1 Key Laboratory of Forage and Endemic Crop Biotechnology, Ministry of Education, School of Life Sciences, Inner Mongolia University, Hohhot 010010, China; starwxz666@163.com (X.W.);; 2 State Key Laboratory of Reproductive Regulatory and Breeding of Grassland Livestock, Inner Mongolia University, Hohhot 010010, China

**Keywords:** halophytes, endophytic fungi, diversity, community structure, functional prediction

## Abstract

Arid desert regions are among the harshest ecological environments on Earth. Halophytes, with their unique physiological characteristics and adaptability, have become the dominant vegetation in these areas. Currently, research on halophytes in this region is relatively limited, particularly concerning studies related to their root endophytic fungi, which have been rarely reported on. Therefore, investigating the diversity and composition of endophytic fungi in halophytes is crucial for maintaining ecological balance in such an arid environment. This study focuses on eight representative angiosperm halophytes from the West Ordos Desert in China (including *Nitraria tangutorum*, *Salsola passerina*, *Suaeda glauca*, *Reaumuria trigyna*, *Reaumuria kaschgarica*, *Limonium aureum*, *Apocynum venetum*, and *Tripolium vulgare*), utilizing Illumina MiSeq high-throughput sequencing technology combined with soil physicochemical factor data to analyze the diversity, composition, and ecological functions of their root-associated fungal communities. Ascomycota dominated the fungal composition in most halophytes, particularly among the recretohalophytes, where it accounted for an average of 88.45%, while Basidiomycota was predominant in *Suaeda glauca*. A Circos analysis of the top 10 most abundant genera revealed *Fusarium*, *Dipodascus*, *Curvularia*, *Penicillium*, and other dominant genera. Co-occurrence network analysis showed significant differences in fungal networks across halophyte types, with the most complex network observed in excreting halophytes, characterized by the highest number of nodes and connections, indicating tighter fungal symbiotic relationships. In contrast, fungal networks in pseudohalophytes were relatively simple, reflecting lower community cohesiveness. Redundancy analysis (RDA) and Mantel tests demonstrated that soil factors such as organic matter, available sulfur, and urease significantly influenced fungal diversity, richness, and evenness, suggesting that soil physicochemical properties play a critical role in regulating fungal–plant symbiosis. Functional predictions indicated that endophytic fungi play important roles in metabolic pathways such as nucleotide biosynthesis, carbohydrate degradation, and lipid metabolism, which may enhance plant survival in saline–alkaline and arid environments. Furthermore, the high abundance of plant pathogens and saprotrophs in some fungal communities suggests their potential roles in plant defense and organic matter decomposition. The results of this study provide a reference for advancing the development and utilization of halophyte endophytic fungal resources, with applications in desert ecosystem restoration and halophyte cultivation.

## 1. Introduction

Arid desert regions are among the most extreme ecological environments on Earth, characterized by persistent water scarcity, irregular and infrequent precipitation, substantial temperature fluctuations, high soil salinity, and limited vegetation cover [1,2]. The increasing severity of global climate change, along with the expansion of human activities and alterations in land use, has accelerated desertification, posing significant challenges to desert ecosystems [3]. Under such extreme conditions, most organisms struggle to survive, with only a few species exhibiting exceptional adaptive capacities. Among these, halophytes dominate these areas due to their unique physiological characteristics and adaptability [4]. Halophytes exhibit remarkable tolerance to salinity, drought, and cold, enabling them to survive and reproduce in environments characterized by high salinity, elevated temperatures, and low water availability [5]. Based on their mechanisms of salt resistance, halophytes are classified into three categories: euhalophytes, pseudohalophytes, and recretohalophytes. Their survival strategies include physiological adaptations such as salt secretion through salt glands, reduced water loss, deep root systems for water uptake, and succulence. These adaptations confer a significant ecological advantage in desert regions [6]. However, these survival strategies not only rely on the plants’ inherent physiological traits but are also closely linked to their interactions with symbiotic microorganisms, particularly endophytic fungi [7].

In halophytes, the diversity and functionality of endophytic fungi play a critical role in enabling plants to withstand drought and high-salinity conditions [8,9]. Halophytes establish symbiotic relationships with these fungi to regulate their metabolic pathways, including osmolyte accumulation and an enhancement in antioxidant enzyme activity, thereby increasing their tolerance to environmental stress [10]. Furthermore, endophytic fungi contribute to the decomposition of organic matter in soil, leading to the release of essential nutrients such as nitrogen, phosphorus, and potassium [11]. These nutrients not only directly nourish plants but also improve soil fertility. Additionally, endophytic fungi secrete phytohormones, such as auxins and cytokinins, which promote root growth and branching, further enhancing the overall growth and resilience of host plants [12,13]. They also participate in plant signaling pathways, inducing defensive responses that enhance the plant’s resistance to pathogens, including systemic acquired resistance (SAR) and induced systemic resistance (ISR). Moreover, endophytic fungi can produce antibiotics and other antimicrobial compounds that inhibit the growth of pathogenic microorganisms, thereby protecting the plant from diseases [14,15].

The Western Ordos region, located in China’s arid and semi-arid zones, is a significant area for biodiversity conservation and serves as a global center for the origin and distribution of temperate grassland shrub species globally. Situated in a transitional zone between grasslands and deserts, this region is shaped by edge effects and paleogeographic factors, resulting in the presence of diverse Mediterranean relict plants as well as rare and endangered desert plants from Central Asia [16,17]. However, research on halophytes in this region remains limited, particularly regarding their root endophytic fungi, which have been scarcely studied. Thus, research on the diversity and composition of endophytic fungi in halophytic plants in this unique arid environment is more important and urgent for maintaining ecological balance. To fill this research gap, we investigated the root-associated endophytic fungal communities of eight representative halophytes in the desert region of Western Ordos, China. The selected halophytes included euhalophytes (*Nitraria tangutorum*, *Salsola passerina*, and *Suaeda glauca*), recretohalophytes (*Reaumuria trigyna*, *Reaumuria kaschgarica*, and *Limonium aureum*), and pseudohalophytes (*Apocynum venetum* and *Tripolium vulgare*) [18]. Using high-throughput sequencing technology combined with soil physicochemical data, the main objectives of this study were as follows: (1) to elucidate the diversity of and compositional changes in endophytic fungi in the roots of different halophytes; (2) to investigate the influence of various soil factors on the structure of endophytic fungal communities; and (3) to predict the functional roles of these endophytic fungi in halophyte roots. The findings of this study contribute to the development and utilization of endophytic fungal resources in halophytes and provide a theoretical basis and reference for the conservation and restoration of desert ecosystems.

## 2. Materials and Methods

### 2.1. Study Site

The study area is located in Wuhai City and its surrounding areas in the West Ordos National Nature Reserve in Inner Mongolia, China. Sampling coordinates range from E 106°45′18.85″ to E 106°53′7.08″ and N 39°32′6.96″ to N 39°51′25.37″ (Figure S1, Table S1), with an average altitude of approximately 1150 m. This site, located at the intersection of the Ulan Buh, Kubuqi, and Maowusu deserts, has a temperate continental climate with a daily mean temperature variation of 13.1 °C and an annual mean of 9.8 °C. The area receives prolonged sunlight, averaging 3227 h of sunshine annually. It is also known for strong winds and frequent sandstorms, with an annual average precipitation of 154.8 mm and an annual evaporation rate of 3343 mm (https://disc.gsfc.nasa.gov/, accessed on 16 August 2023), more than 20 times the annual precipitation, resulting in an arid climate and severe soil desertification. The main soil types in the area include gray desert soil, aeolian sandy soil, brown calcic soil, and saline soil. The dominant vegetation types are psammophytic vegetation, desertified grassland vegetation, desert vegetation, and arid grassland vegetation, with xerophytic shrubs and semi-shrubs occupying a dominant ecological position in plant communities [19,20].

### 2.2. Sample Collection

In this study, 24 samples were collected from eight angiosperm species of halophytes, categorized into three groups: euhalophytes including *Nitraria tangutorum* (Nt), *Salsola passerina* (Sp), and *Suaeda glauca* (Sg); recretohalophytes comprising *Reaumuria trigyna* (Rt), *Reaumuria kaschgarica* (Rk), and *Limonium aureum* (La); and pseudohalophytes including *Apocynum venetum* (Av) and *Tripolium vulgare* (Tv) [18]. These species were selected for their distinct ecological strategies in saline and arid environments, representing key halophyte types with different salt tolerance mechanisms. The abbreviations in parentheses are used throughout this text to represent these species. To minimize the impact of plant age on the root endophytic fungal communities and their structure, three 10 × 10 m plots were established for each plant species, to account for variability in fungal communities. Three root samples were collected from each plot, resulting in nine samples per species. These nine samples were then homogenized to create three composite samples, yielding a total of twenty-four samples across the eight species. This replication was deemed adequate for statistical analysis. The root samples were immediately placed into cryovials, transferred to a liquid nitrogen tank, and stored at −80 °C until they were returned to the laboratory for subsequent high-throughput sequencing analysis. Simultaneously, rhizosphere soil samples were collected using disposable brushes, creating three composite soil samples per plant species. These soil samples were stored in an insulated container at 4 °C during transportation to the laboratory, where they were further preserved at 4 °C for the subsequent analysis of soil physicochemical properties.

### 2.3. Soil Physical and Chemical Determination

The soil pH was measured by a soil monitoring instrument (SKW500), while soil moisture content was determined using the drying method. Ammonium nitrogen (NH_4_^+^-N) and nitrate nitrogen (NO_3_^−^-N) were quantified with an automatic Kjeldahl nitrogen analyzer. Organic matter content was assessed via the dichromate oxidation method. Soil catalase (CAT) activity was measured by the potassium permanganate titration method, and soil urease (UE) activity was evaluated using the sodium phenolate–sodium hypochlorite colorimetric method. Soil sucrase (SC) activity was determined by the 3,5-dinitrosalicylic acid colorimetric method. Total phosphorus (TP) and alkaline phosphatase (ALP) were measured using spectrophotometry [21]. Inorganic phosphorus (Pi), available potassium (Ak), available sulfur (Es), and cellulase (CL) activities were determined using commercial assay kits (Shanghai YuXuan Biotechnology Co., Ltd. Shanghai, China) following the manufacturer’s instructions. Additionally, all measured samples were subjected to three biological replicates.

### 2.4. DNA Extraction and PCR Amplification

Following the manufacturer’s instructions, DNA was extracted from composite samples using a DNA extraction kit (MoBio Laboratories, Carlsbad, CA, USA), producing three aliquots of DNA. DNA amplification was performed using an ABI GeneAmp^®^ Model 9700 PCR system. The quality of the DNA extraction was assessed by 1% agarose gel electrophoresis (5 V/cm, 20 min), and DNA concentration and purity were evaluated using a NanoDrop^®^ 2000 spectrophotometer (Thermo Scientific, Wilmington, DE, USA). The fungal ITS regions were amplified using the primers ITS1 (5′-TCCGTAGGTGAACCTGCGG-3′) and ITS4 (5′-TCCTCCGCTTATTGATATGC-3′). The PCR mixture (50 µL) consisted of 25 µL 2× Taq PCR Master Mix, 2 µL each of primers ITS1 and ITS4, 2 µL DNA template, and ddH_2_O for a final volume of 50 µL. The PCR amplification program was as follows: initial denaturation at 94 °C for 5 min, followed by 35 cycles of denaturation at 94 °C for 30 s, annealing at 55 °C for 40 s, and extension at 72 °C for 1 min, with a final extension at 72 °C for 3 min. PCR products were recovered from 2% agarose gel electrophoresis and purified using an AxyPrep DNA Gel Extraction Kit (Axygen Biosciences, Union City, CA, USA). The quantification of the purified PCR products was conducted using a Quantus Fluorometer (Promega, Madison, WI, USA).

### 2.5. Illumina Sequencing and Bioinformatics Analysis

Community DNA fragments were sequenced in paired-end mode using the Illumina MiSeq platform (Illumina, San Diego, CA, USA). Primers were trimmed from the sequences using the qiime cutadapt trim-paired function in QIIME2 software, and sequences without matching primers were discarded. Subsequently, quality control, denoising, merging, and chimera removal were performed using the qiime dada2 denoise-paired function, leveraging the DADA2 algorithm. These steps were conducted separately for each library. After denoising all libraries, ASV (Amplicon Sequence Variant) feature sequences and ASV tables were merged, singletons were removed, and the feature table was rarefied to 95% of the minimum sample sequence depth using the qiime feature table rarefy function. The length distribution of sequences was also assessed. Fungal ITS sequences were taxonomically classified using the UNITE database (Release 8.0, https://unite.ut.ee, accessed on 9 May 2023). Finally, alpha diversity metrics were used to analyze and characterize the richness, evenness, and diversity of the endophytic fungal communities.

### 2.6. Statistical Analyses

The statistical analyses in this study were performed using R software version 3.3.1, while a one-way analysis of variance (ANOVA) was conducted with IBM SPSS Statistics version 26.0, with significance thresholds set at * *p* < 0.05 and ** *p* < 0.01. Community analyses were performed using petal diagrams and Venn diagrams based on ASV abundance tables. To explore potential biomarkers at different taxonomic levels, linear discriminant analysis (LDA, score > 4.0) combined with effect size (LEfSe) was employed (http://huttenhower.sph.harvard.edu/galaxy/, accessed on 17 May 2023). The R package “Circlize” was used to create Circos plots to determine associations between dominant phyla or genera and halophyte sample groups. Non-metric multidimensional scaling (NMDS) and hierarchical clustering, both using the Bray–Curtis distance algorithm, were applied to evaluate the distribution characteristics of endophytic fungi. To construct a network of dominant species, the induced subgraph function in the R package “igraph” was utilized based on ASV abundance, and the topological features of the co-occurrence network were computed using the same package. Gene sequence prediction was conducted with PICRUSt2 using the MetaCyc functional database (https://metacyc.org/, accessed on 19 June 2023), and fungal community functions were predicted with FUNGuild. The R packages “ggplot2” and “linkET” were employed for Mantel tests and Pearson correlation analyses to examine the relationships between endophytic fungal communities and soil factors. Redundancy analysis (RDA) was also performed to determine the association between fungal community composition and soil-related factors. The data from physicochemical measurements were normalized before conducting the redundancy analysis.

## 3. Results

### 3.1. Sequencing Data and Taxonomic Statistics of Endophytic Fungi

**Figure 1:**
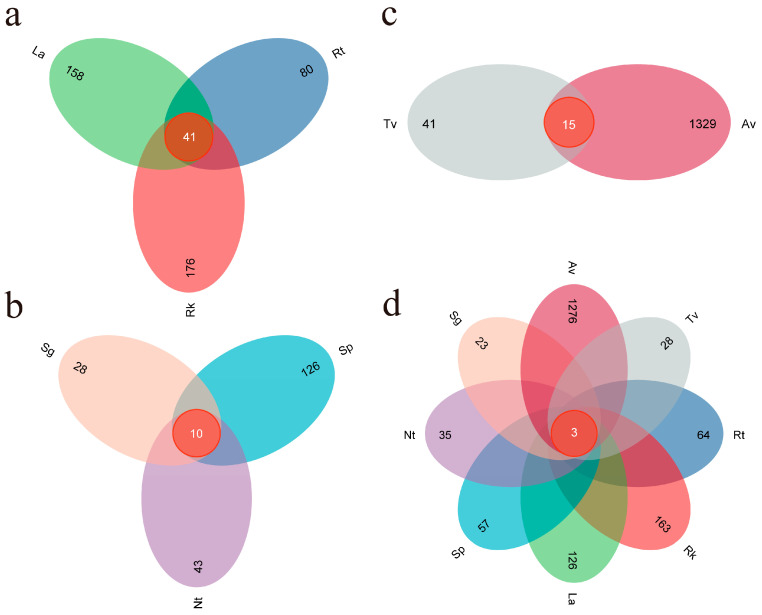
Clustering and comparative analyses of Amplicon Sequence Variants (ASVs) in endophytic fungi from different halophytes: (**a**) recretohalophytes, (**b**) euhalophytes, (**c**) pseudohalophytes, and (**d**) represents all eight types of halophytes.

The high-throughput sequencing of eight different types of halophytes produced a total of 3,329,762 raw sequences. After the quality filtering, denoising, and merging of reads from different samples, 2,811,041 high-quality reads were retained, resulting in a total of 1974 fungal Amplicon Sequence Variants (ASVs). Rarefaction curves calculated using different indices for all root samples indicated that the ASV curves reached a plateau (Figure S2), suggesting sufficient sequencing depth and reliable data. Clustering and comparative analyses of the 1974 fungal ASVs revealed that only 3 ASVs were shared among the eight plant species, indicating a high degree of specificity in the root-associated fungal communities. Av had the highest number of unique ASVs (1276), while Sg had the fewest (23), possibly due to the more favorable root environment of Av for diverse fungal communities (Figure 1d). Additionally, the numbers of ASVs shared among recretohalophytes, euhalophytes, and pseudohalophytes were 41, 10, and 15, respectively (Figure 1a–c). These results indicate that a high degree of specificity in root-associated fungal communities underscores the ecological uniqueness of each halophyte species in harboring specialized fungal taxa.

### 3.2. Diversity of Endophytic Fungi

**Figure 2:**
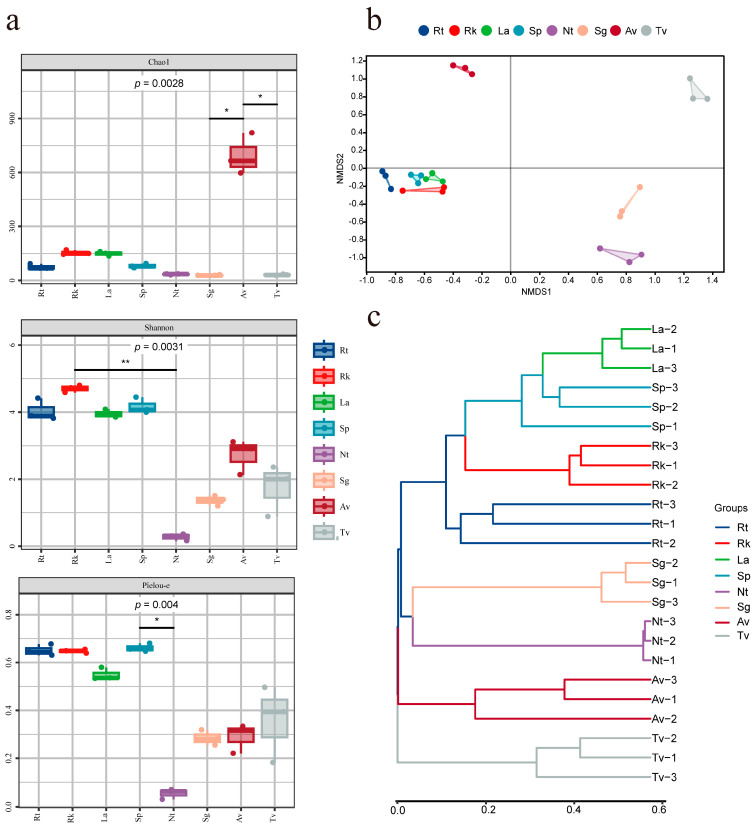
Analysis of endophytic fungal diversity in eight halophyte species. (**a**) Alpha diversity index. (**b**) NMDS analysis (non-metric multidimensional scaling analysis). (**c**) Cluster analysis based on Bray–Curtis distance algorithm. Wilcoxon rank-sum test was used to analyze *p* value. * represents significant difference between mean values of two groups of samples. Significance levels are as follows: * *p* < 0.05, ** *p* < 0.01.

The diversity of endophytic fungi in the roots of eight halophyte species was assessed using alpha diversity metrics, including the Chao1 index for richness, the Shannon index for diversity, and Pielou’s evenness index for evenness (Figure 2a, Table S2). According to the Chao1 index, Av exhibited the highest richness (693.67), while Sg and Tv showed relatively low richness values (28.57 and 30.52, respectively), both significantly differing from Av (*p <* 0.05). Shannon diversity analysis revealed that all recretohalophytes and the euhalophyte Sp had high diversity indices, all exceeding 4. In contrast, Nt showed a low diversity with a Shannon index of only 0.27, significantly differing from all plants except Rt (*p <* 0.01). Among the pseudohalophytes, the Shannon indices for Av and Tv were 2.72 and 1.75.

A significant variation in evenness (Pielou’s index) was observed among the three euhalophytes, particularly between Sp and Nt (*p* < 0.05). This suggests that the root environment of Nt supports a limited range of fungi, whereas Sp provides a more favorable habitat for diverse fungal species. NMDS analysis demonstrated a clear separation of endophytic fungal communities among different halophytes (Figure 2b). Interestingly, the diversity of Sp among the euhalophytes was closer to that of the recretohalophytes. Clustering analysis based on the Bray–Curtis distance algorithm (Figure 2c) supported similar conclusions. These results suggest that environmental and physiological factors, such as root habitat conditions, soil salinity, and plant growth strategies, may contribute to the higher diversity observed in some halophytes compared to others.

### 3.3. Community Composition of Endophytic Fungi

**Figure 3:**
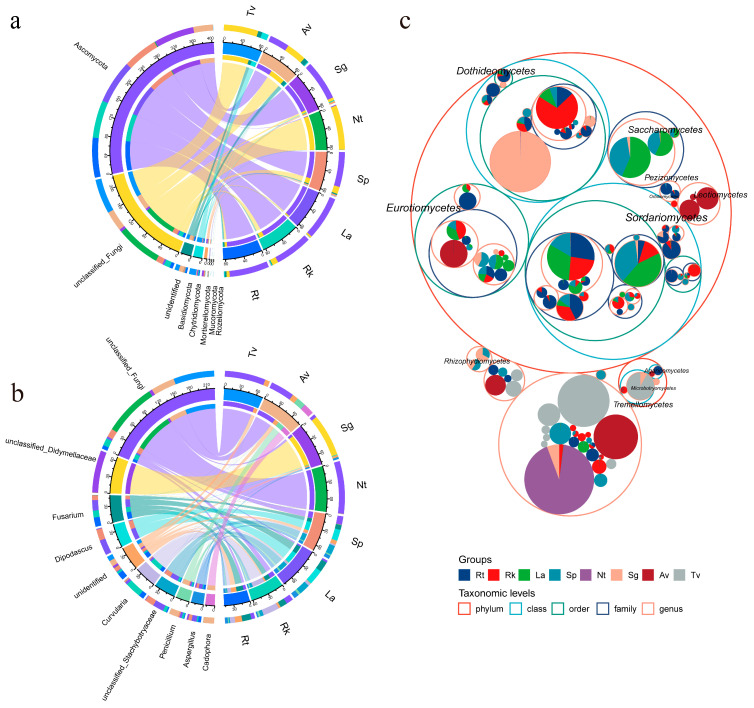
Overlapping and divergent endophytic fungal taxa in eight halophyte groups. (**a**) Representative phylum level, (**b**) representative genus level, (**c**) classification level tree.

A circular visualization chart illustrated the overlap and differentiation of endophytic fungal taxa at the phylum and genus levels among the eight plant groups (Figure 3a,b). The most abundant phyla observed across all halophyte groups were Ascomycota, Basidiomycota, Chytridiomycota, and some unclassified fungi. Ascomycota was the dominant phylum in all plants except for Tv, particularly in recretohalophytes (Rt, Rk, and La), where it averaged 88.45%, and in euhalophytes, Sg and Sp, with proportions exceeding 75%. In contrast, Basidiomycota dominated in the pseudohalophyte Tv (Figure 3a). A Circos analysis of the top 10 most abundant genera revealed *Fusarium*, *Dipodascus*, *Curvularia*, and *Penicillium* as dominant genera (Figure 3b). *Fusarium* was prevalent in Rt, Rk, La, and Sp, while *Curvularia* dominated in Rk (30.24%) and *Dipodascus* in La and Sp (28.64% and 20.93%, respectively). In the pseudohalophytes, Av and Tv, the dominant genera were *Cadophora* (20.27%) and *Filobasidium* (14.97%), respectively. The dominance of genera such as *Fusarium*, *Curvularia*, and others suggests they may have a potential role in plant stress tolerance or nutrient cycling, warranting further investigation.

A taxonomic rank tree was constructed to visualize the distribution of fungal taxa (Figure 3c), where the largest circle represents the phylum level, with progressively smaller circles for class, order, family, and genus. At the class level, Sordariomycetes were enriched in the three recretohalophytes and in the euhalophyte Sp. Saccharomycetes was also highly represented in La and Sp, while Dothideomycetes had a particularly high proportion in the euhalophyte Sg. In the pseudohalophytes, Leotiomycetes and Eurotiomycetes dominated in Av, and Tremellomycetes was the dominant class in Tv.

### 3.4. LEfSe Analysis of Dominant Taxa of Endophytic Fungi

**Figure 4:**
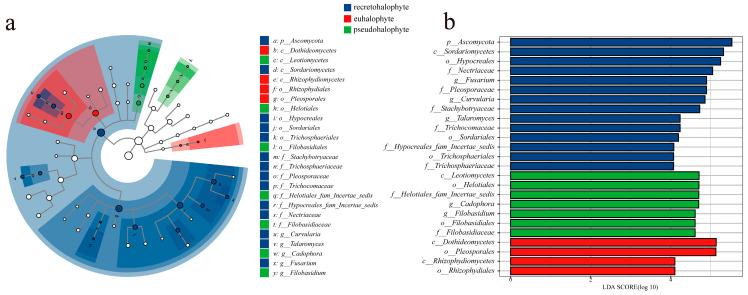
LEfSe analysis of abundance of endophytic fungi in eight halophyte species. (**a**) Cladogram is used to show taxonomic distribution of marker species in each group of samples, (**b**) indicator microbial groups with LDA scores > 4.

LEfSe analysis revealed significant differences in endophytic fungal communities across 25 taxonomic levels within the roots of three different types of halophytes, with an LDA score set at 4.0 (Figure 4). Compared to euhalophytes and pseudohalophytes, the endophytic fungal community of recretohalophytes displayed the greatest number of evolutionary branches, totaling 14, including 1 phylum, 1 class, 3 orders, 6 families, and 3 genera. Specifically, the phylum level is predominantly Ascomycota, encompassing the class Sordariomycetes. At the order level, the communities are primarily enriched in Hypocreales, Sordariales, and Trichosphaeriales; at the family level, notable biomarkers include Stachybotryaceae, Trichosphaeriaceae, Pleosporaceae, and Trichocomaceae; and at the genus level, significant groups are *Curvularia*, *Talaromyces*, and *Fusarium*.

In contrast, the fungal community of pseudohalophytes is enriched with seven biomarkers, including the class Leotiomycetes (which is further subdivided into the orders Helotiales, the family Helotiales_fam_Incertae_sedis, and the genus *Cadophora*), as well as the order Filobasidiales, the family Filobasidiaceae, and the genus *Filobasidium*. Euhalophytes were characterized by only four significant taxa: Dothideomycetes (class), Rhizophydiomycete (class), Rhizophydiales (order), and Pleosporales (order). The differences in fungal communities among different types of halophytes are likely closely related to their adaptation mechanisms. Future research should further investigate how these differences impact plant stress tolerance and adaptability.

### 3.5. Co-Occurrence Network of Endophytic Fungi

**Figure 5:**
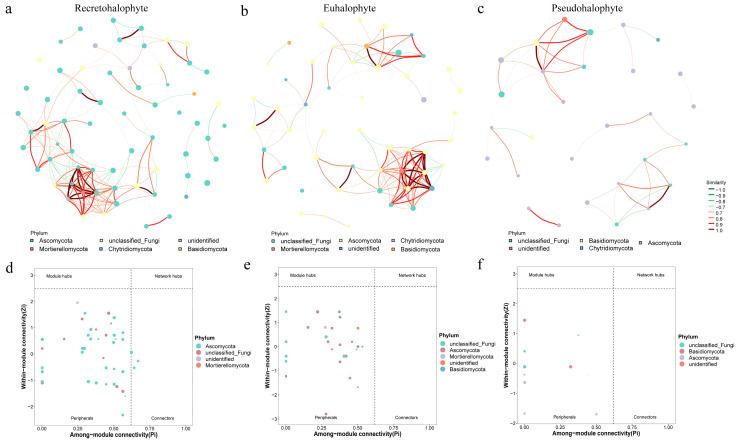
Co-occurrence network pattern of three different types of halophytes. (**a**–**c**) represent recretohalophytes, euhalophytes, and pseudohalophytes, respectively. (**d**–**f**) represent Zi-Pi graphs based on topological roles in networks of recretohalophytes, euhalophytes, and pseudohalophytes, respectively.

A co-occurrence network analysis of endophytic fungal communities in the roots of three halophyte types revealed significant differences in interaction patterns (Figure 5a–c). The modularity of the random networks was significantly lower than that of the empirical networks, suggesting that the empirical networks had a better structure. The average path length ranged from 2.29 to 4.10, indicating that these fungal communities tend to form clusters, with a small-world network structure [22,23]. The co-occurrence network patterns among the three types of halophytes showed significant differences. Core endophytic fungi produced 65, 51, and 29 nodes, with 197, 135, and 41 connections in recretohalophytes, euhalophytes, and pseudohalophytes, respectively (Table S3). Compared to the other two types, recretohalophytes exhibited a higher number of vertices and edges. Additionally, the average nearest neighbor degree for all nodes in the recretohalophyte network was the highest, indicating a more complex endophytic fungal community structure. In terms of transitivity, euhalophytes showed better transitivity (0.72), while recretohalophytes and pseudohalophytes had transitivity values of 0.60 and 0.57, respectively.

By calculating within-module connectivity (Zi) and between-module connectivity (Pi), we identified potential core species (Figure 5d–f). Ascomycota emerged as a key group across all three types of halophytes. Additionally, Mortierellomycota was found to be significant in both recretohalophytes and euhalophytes, while Basidiomycota was prominent in euhalophytes and pseudohalophytes. A further analysis of the co-occurrence network revealed that the core endophytic fungal community of recretohalophytes predominantly resides in peripheral clusters, with only a small portion in connector clusters. In contrast, the core endophytic fungal communities of the other two halophytes were entirely classified as peripheral clusters. In summary, recretohalophytes are the most complex, reflecting a higher degree of symbiotic relationships. Euhalophytes exhibit the highest network transitivity, indicating stronger connectivity, while pseudohalophytes show the lowest network complexity, with a relatively simpler fungal community structure. Higher modularity in recretohalophytes suggests more distinct and potentially functionally specialized fungal interactions compared to the other groups.

### 3.6. Relationship Between Endophytic Fungi and Soil Factors

**Figure 6:**
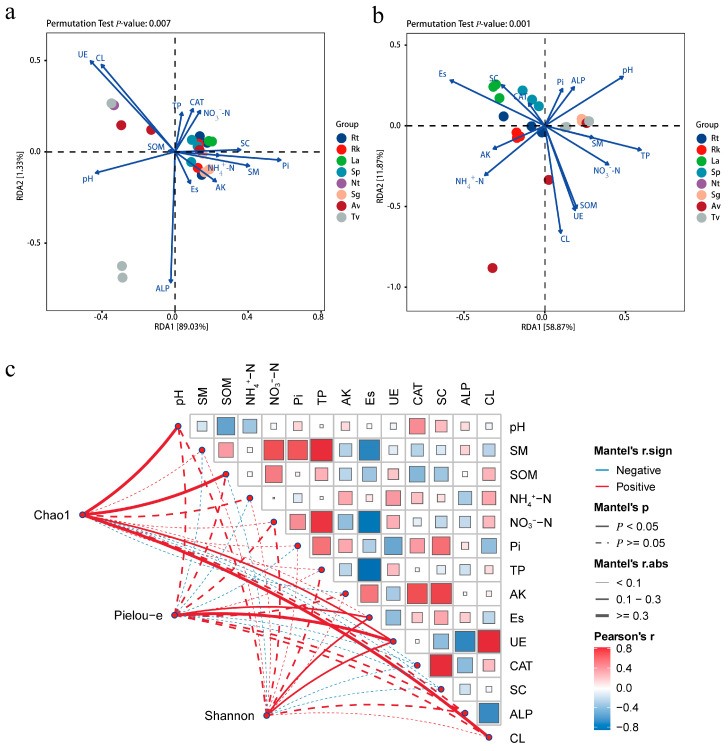
An RDA plot (redundancy analysis plot). (**a**,**b**) represent the associations between endophytic fungi in different halophytes and soil factors at the phylum and genus levels, respectively. (**c**) represents the Mantel correlation analysis between soil physicochemical factors and endophytic fungal diversity. The line color represents a significant difference. The thickness of the line represents the size of the correlation coefficient.

This study also collected rhizosphere soil samples from plant roots to investigate the influence of soil-related factors on the endophytic fungal communities of halophytes. The results from RDA and Mantel tests indicated that the composition of these fungal communities was correlated with various soil physicochemical factors (Figure 6). RDA at the phylum level (Figure 6a) revealed that the RDA1 and RDA2 axes explain 1.33% and 89.03% of the variance in the structure of endophytic fungal communities in halophyte roots, respectively, for a total of 90.36%. Among these factors, alkaline phosphatase (ALP), urease (UE), and cellulase (CL) have significant impacts on fungal diversity. A further RDA of the top 10 dominant genera within endophytic fungal communities showed that the RDA1 and RDA2 axes explained 11.87% and 58.87% of the variance, respectively, totaling 70.74% (Figure 6b). Effective sulfur (ES), cellulase (CL), and pH exhibited strong correlations, with higher explanatory power, highlighting the relationship between endophytic fungi and soil physicochemical factors at both the phylum and genus levels.

The Mantel test results indicated (Figure 6c) that soil organic matter (SOM) significantly affected Chao1 richness, with a correlation coefficient of 0.48 (*p* < 0.05). Additionally, pH (r = 0.32), urease (UE) (r = 0.19), and cellulase (CL) (r = 0.34) also showed significant positive correlations with fungal richness (*p* < 0.05) (Figure 6c). In contrast, Shannon diversity and Pielou’s evenness were significantly influenced by effective sulfur (ES) and urease (UE) (*p* < 0.05), with correlation coefficients of 0.30 and 0.23 for ES and 0.28 and 0.35 for UE, respectively. These results emphasize the key role of soil physicochemical factors, particularly nutrient cycling enzymes like alkaline phosphatase and cellulase, in shaping the composition and diversity of endophytic fungal communities in halophytes.

### 3.7. Functional Prediction of Endophytic Fungi

**Figure 7:**
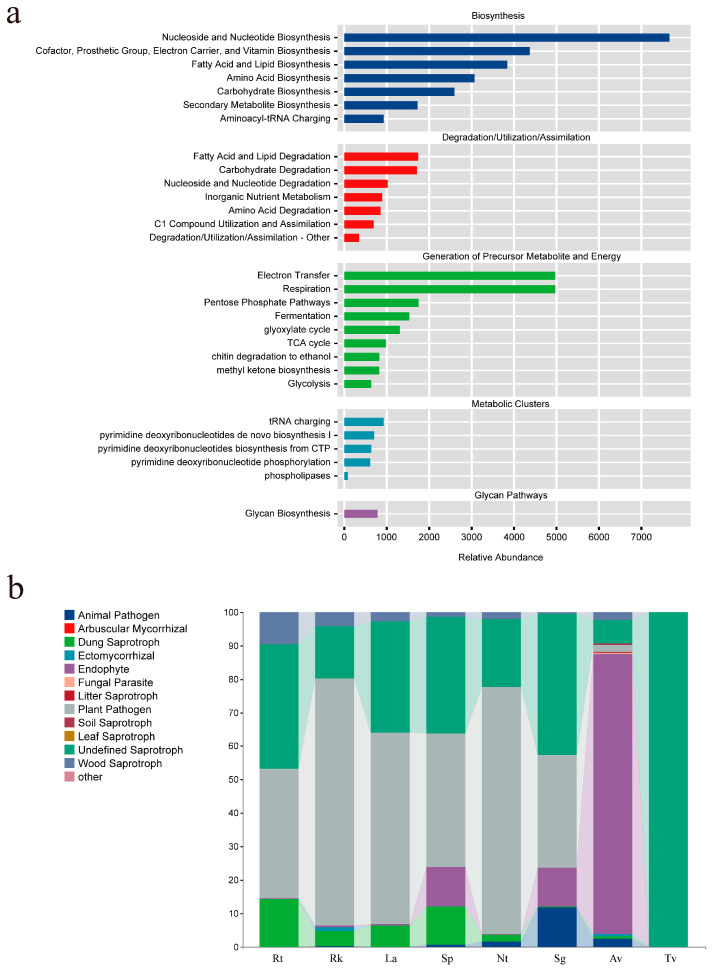
(**a**) shows the relative abundance of endophytic fungal metabolic pathways based on PICRUSt2 analysis, while (**b**) represents the functional prediction of endophytic fungi using FUNGuild.

This study employed PICRUSt2 to predict the functional profiles of endophytic fungi in eight halophyte species (Figure 7a). An analysis of metabolic pathways from the MetaCycle database revealed five primary metabolic categories: Biosynthesis, Degradation/Utilization/Assimilation, Generation of Precursor Metabolite and Energy, Glycan Pathways, and Metabolic Clusters. At the second level, 29 metabolic pathways were enriched. In the Biosynthesis category, seven pathways were annotated, with Nucleoside and Nucleotide Biosynthesis exhibiting the highest abundance. The Degradation/Utilization/Assimilation category also included seven pathways, with Carbohydrate and Fatty Acid Degradation showing higher abundance. For the Generation of Precursor Metabolite and Energy, nine pathways were annotated, with Electron Transfer and Respiration being the most abundant. Glycan Pathways were enriched in Glycan Biosynthesis. Finally, within Metabolic Clusters, tRNA charging was the most enriched pathway.

Additionally, this study used FUNGuild to predict the functions of endophytic fungi (Figure 7b). In the three recretohalophytes, plant pathogens were found to be the predominant functional guild, followed by undefined saprotrophs, dung saprotrophs, and wood saprotrophs, which also exhibited relatively high abundances. In euhalophytes, plant pathogens and undefined saprotrophs were consistently abundant across all three plant species, similar to the results observed for recretohalophytes. Furthermore, a notable presence of endophytes was found in Sp and Sg, with Sg showing a higher abundance of Animal Pathogens. In pseudohalophytes, a significant presence of undefined saprotrophs was observed in Av, but endophytes were the predominant group in Av. In contrast, undefined saprotrophs were extremely prevalent in Tv. The predominance of plant pathogens in recretohalophytes suggests potential antagonistic interactions, whereas the higher abundance of endophytes in pseudohalophytes may indicate mutualistic relationships beneficial for stress adaptation.

## 4. Discussion

The structure of endophytic fungal communities is influenced by various biotic and abiotic factors, including climate, host identity, and biological or environmental stressors. Among these factors, host identity is a major determinant of the composition of endophytic fungal communities. This study found that the dominant fungal communities in the roots of eight halophyte species from the West Ordos Desert shared similar compositions. This similarity suggests that, under comparable saline–alkaline conditions, these plants may selectively attract and maintain certain beneficial fungi to help them cope with adverse conditions. Although the dominant fungal communities are similar, their relative abundances vary, and the composition of low-abundance fungal groups differs as well. These variations reflect the specific needs and selective pressures by different plants on their root microbiota, which may be influenced by the unique physiological characteristics and ecological strategies of each plant.

### 4.1. Endophytic Fungal Diversity in Halophytes

This study analyzes the richness, evenness, and diversity of endophytic fungi in the roots of eight halophyte species. It reveals differences in the structure of fungal communities among different types of halophytes. According to the Chao1 index, *Apocynum venetum* exhibits significantly higher richness of endophytic fungi compared to *Suaeda glauca*, with a difference of approximately 24 times. As a euhalophyte, *Suaeda glauca* adapts to high-salinity environments by accumulating salts within its tissues, particularly in its leaves and stems, through salt compartmentalization. This strategy helps to reduce the internal salt load of the plant. However, the salt accumulation in the tissues also leads to high salt concentrations in the root zone, which can hinder the survival of most endophytic fungi [6,24]. In contrast, *Apocynum venetum*, a pseudohalophyte and perennial shrub, employs specialized mechanisms such as selective ion uptake and exclusion by its roots, along with possibly salt-secreting glands or root exudates, to limit salt accumulation in its tissues and root zone. These adaptations maintain a lower salt concentration in the rhizosphere, creating a more favorable environment for diverse endophytic fungi, thereby promoting higher species richness [6,25]. Conversely, *Tripolium vulgare* (another pseudohalophyte, but an annual herb) has a lower richness of root fungi, possibly due to its shorter life cycle [26].

The Shannon index, which integrates both species richness and evenness, reveals that *Reaumuria kaschgarica*, as a recretohalophyte, can expel salts externally and restrict the accumulation of salts in non-essential tissues (such as vacuoles or external layers), maintaining a low-salinity environment in the root zone, fostering a more diverse and balanced endophytic fungal community [6]. This phenomenon is consistent with the findings of Wang et al., which suggested that while salinity has a minimal impact on recretohalophytes and pseudohalophytes, it significantly affects euhalophytes [27]. Although *Salsola passerina* is also a euhalophyte, its endophytic fungal diversity remains higher than that of other euhalophytes (Figure 2a). This difference may be attributed to several factors such as the unique root salinity distribution, physiological adaptations, rhizosphere microenvironment, and genetic diversity of *Salsola passerine*, which need to be proven in future studies [28]. The above study suggests a possible co-evolutionary relationship between halophytes and their endophytic fungi, which may influence fungal diversity in response to environmental pressures.

### 4.2. Endophytic Fungal Community Structure in Halophytes

An analysis of the endophytic fungal communities in the roots of eight halophyte species revealed that they were primarily dominated by Ascomycota, Basidiomycota, and Chytridiomycota, with limited diversity at the phylum level. This limited diversity may be largely attributed to the nutrient-poor soils of saline–alkali desert regions in arid areas. Zuo’s research indicates that Ascomycota dominates the fungal communities in shrub plants found in extremely arid regions [29]. Similarly, this study found that, except for *Tripolium vulgare*, the root endophytic fungi of the other seven halophyte species were dominated by Ascomycota, consistent with previous findings. However, in *Tripolium vulgare*, Basidiomycota was the dominant phylum, distinguishing it from the other halophytes. Previous studies have demonstrated the Ascomycota and Basidiomycota differ in their ecological adaptability [30].

Ascomycota, the most abundant and widely adaptable phylum, is capable of decomposing recalcitrant organic materials such as plant fibers and lignin, allowing it to thrive in early-stage, nutrient-poor soil environments [31,32]. In contrast, Basidiomycota is better adapted to moist environments and tends to be more dominant in later successional soil stages [33]. Consequently, in arid desert environments, where resource competition is intense, Ascomycota tends to be more competitive. Although *Tripolium vulgare* is commonly found in saline–alkali soils, it is also frequently observed in more humid environments, such as marshes, lakeshores, and coastlines [34]. It is hypothesized that through years of evolution, the roots of *Tripolium vulgare* have become more conducive to the presence of Basidiomycota in such moist environments. This may explain why Basidiomycota dominates the endophytic fungal communities in the roots of *Tripolium vulgare*. Future studies could compare the endophytic fungal diversity and Basidiomycota abundance in *Tripolium vulgare* roots under arid and moist conditions to test this hypothesis. Additionally, since both Ascomycota and Basidiomycota are saprophytic fungi, we hypothesize that allowing more saprophytic fungi to exist in desert plants could maximize the potential for in situ nutrient recovery and promote nutrient cycling in desert soils [35].

Through an investigation of endophytic fungi in various halophyte plants at the class level, we found that *Sordariomycetes* were highly enriched in three recretohalophytes and *Salsola passerina*. Many species within *Sordariomycetes* are important decomposers of wood and plant debris, capable of breaking down complex organic materials, including genera such as *Curvularia*, *Talaromyces*, and *Fusarium* [36]. Moreover, *Sordariomycetes* may promote plant growth by inducing defensive responses and enhancing plant resilience to environmental stressors such as salinity and drought [37]. In some euhalophytes, *Dothideomycetes* were found to be dominant. *Dothideomycetes* can provide essential nutrients, such as nitrogen and phosphorus, thereby facilitating plant growth in high-salinity environments [38]. In the pseudohalophyte *Apocynum venetum*, *Eurotiomycetes* were the most abundant class. Although some species within *Eurotiomycetes* are known plant pathogens, they may indirectly protect the host plant in specific ecosystems by competitively suppressing other more aggressive pathogens [39,40]. This study also found that *Saccharomycetes* were present in significant proportions in *Limonium aureum* and *Salsola passerina*. *Saccharomycetes* primarily consist of yeast species that play a crucial role in sugar fermentation and metabolism [41]. In halophyte plants, these yeasts may help regulate osmotic pressure within the plant through their metabolic activities, thereby aiding in adaptation to saline–alkaline environments. Additionally, *Saccharomycetes* may be involved in nutrient cycling and disease resistance mechanisms within plants [42].

LEfSe analysis confirmed variations in endophytic fungal community structures across different halophyte plants, suggesting that endophytic fungi exhibit host-specific preferences. These differences may result from varying microbial life histories, species pools, and ecological niches [43]. In the co-occurrence network, further analysis revealed that the core endophytic fungal communities of recretohalophytes were predominantly composed of peripheral clusters, with only a small portion appearing in connector clusters. In contrast, the core fungal communities of both euhalophytes and pseudohalophytes consisted entirely of peripheral clusters. This suggests that the endophytic fungal community structure of recretohalophytes contains certain fungi that play a critical role in linking different modules, while the fungal communities of euhalophytes and pseudohalophytes are primarily made up of peripheral fungi. It can be inferred that the small number of connector fungi in recretohalophytes may play a vital role in facilitating interactions and material exchange among distinct fungal communities, thereby enhancing the overall stability and functionality of the fungal network [44,45].

### 4.3. Association Between Endophytic Fungal Diversity and Soil Factors

Previous studies have demonstrated that soil physicochemical factors significantly influence the assembly of endophytic fungal communities by regulating the availability of soil nutrients and chemical properties, such as pH, enzyme activity, and organic matter content, thereby determining the survival and reproduction of endophytic fungi [46]. Our study revealed that alkaline phosphatase, urease, and cellulase have a significant impact on the diversity of endophytic fungi in halophyte roots.

Alkaline phosphatase enhances the availability of phosphorus in soil by mineralizing organic phosphorus [47], which endophytic fungi can absorb to increase their reproductive capacity. Urease decomposes urea in soil, releasing nitrogen, an essential nutrient for both plants and fungi. By breaking down urea, urease increases the availability of nitrogen in soil [48], thereby supporting the metabolic activities of endophytic fungi. Additionally, previous studies have shown that carbon is a primary energy source and building block for microorganisms. Cellulase facilitates the release of carbon sources by decomposing organic matter in soil [49]. Our study found that organic matter is the most significant factor influencing the Chao1 richness of endophytic fungi. Higher organic matter content correlates with greater fungal diversity, possibly due to the provision of abundant carbon sources that support a more diverse microbial community. Moreover, the activities of these enzymes not only directly affect soil nutrient availability but also indirectly promote the symbiotic relationship between endophytic fungi and host plants by improving soil structure.

Additionally, our study found a positive correlation between available sulfur and both the diversity and evenness of endophytic fungi. Sulfur is an essential element for the growth of both microorganisms and plants, serving multiple critical roles. On one hand, sulfur is a component of various amino acids, such as cysteine and methionine, which are fundamental building blocks of proteins. On the other hand, sulfur also plays a key role in enzyme synthesis. Many enzymes contain sulfur groups; for example, thiol-containing enzymes (such as glutathione peroxidase) use sulfur groups to perform redox reactions, protecting cells from oxidative stress. Therefore, the availability of sulfur may directly influence the structure of these fungal communities [50]. In addition to these factors, other soil parameters, such as salinity levels and the availability of micronutrients, may also play a critical role in fungal community assembly. Future studies could further investigate the effects of these parameters on the assembly of endophytic fungi in halophytes.

### 4.4. Functional Prediction of Endophytic Fungi in Halophytes

Metabolic pathway analysis using the MetaCycle database in PICRUSt2 reveals several significant functions of endophytic fungi in different halophytic plants [51]. The high abundance of pathways related to nucleoside and nucleotide biosynthesis suggests that these fungi may play a critical role in supporting rapid plant growth and repair. Additionally, the emphasis on carbohydrate degradation and fatty acid/lipid degradation highlights the importance of endophytic fungi in supplying essential carbon sources and energy, benefiting both the fungi and plants [52]. This observation is consistent with our findings that cellulase and organic matter significantly influence the structure of endophytic fungal communities, highlighting the pivotal role of carbon sources in both endophytic fungal and plant ecosystems. The essential roles of electron transfer and respiration in energy metabolism indicate that endophytic fungi may enhance plant stress resistance by ensuring a stable energy supply. Furthermore, the enrichment of pathways related to glycan biosynthesis suggests that endophytic fungi may assist plants in cell wall construction and signal transduction. Previous studies have demonstrated that tRNA is crucial in protein synthesis [53], and this study hypothesizes that endophytic fungi may enhance the growth of halophytic plants and their ability to adapt to saline–alkali environments by optimizing protein synthesis pathways.

This study also utilized FUNGuild to predict the functional roles of endophytic fungi. Despite the differences in the structures of endophytic fungal communities across various types of halophytic plants, there was a consistently high abundance of plant pathogens and undefined saprotrophs. In recretohalophytes and euhalophytes, the high abundance of plant pathogens may exert disease pressure on plants, potentially driving the evolution of more robust defense mechanisms [54]. The significant abundance of undefined saprotrophs indicates their significant role in organic matter decomposition and nutrient cycling [55,56]. The presence of endophytes, particularly notable in euhalophytes and pseudohalophytes, suggests that these fungi may promote plant growth and enhance stress resistance through symbiotic relationships. In conclusion, the nutrient cycling functions of fungi may contribute to the overall resilience of desert ecosystems by improving soil nutrient availability, enhancing water retention, and supporting plant adaptation to climate change. Although this study provides insights into the potential functions of endophytic fungi, these remain speculative, particularly regarding their contributions to plant growth, stress tolerance, and the symbiotic relationship between fungi and plants. Future research should focus on the experimental validation of these hypotheses.

At the same time, we will also focus on the following areas in future research: investigating how seasonal and environmental variations influence the diversity and composition of endophytic fungal communities in halophytes, whether fungi possess genetic adaptations that enable their survival in extreme saline–alkaline environments, and the role of bacterial communities in conjunction with fungi in shaping plant health and enhancing stress resilience.

## 5. Conclusions

This study reveals the crucial role of root-associated endophytic fungi, particularly members of Ascomycota and Basidiomycota, in arid desert ecosystems. These fungi not only enhance the resistance, adaptability, and nutrient acquisition capabilities of halophytes but also promote plant survival and ecosystem stability in extreme environments through complex ecological networks. Mechanisms such as salt detoxification, nutrient cycling, and the modulation of the host plant’s physiological processes play an important role in this process. The diversity and community structure of endophytic fungi in different halophyte species reflect their distinct ecological adaptation strategies. These fungi have significant potential in practical applications, particularly in the development of biofertilizers and plant biostimulants for agriculture in saline–alkali soils. Future research should utilize techniques such as genome sequencing, metabolomics, and field trials to further investigate the interactions between these fungi and plants, providing practical guidance for the conservation and restoration of desert ecosystems.

## Data Availability

All available data are published in this paper and the Supplementary Materials.

## Supplementary Materials

The following supporting information can be downloaded at https://www.mdpi.com/article/10.3390/jof11010030/s1, Table S1: Basic spatial geographic parameter information of study site. Table S2: Alpha diversity indices of six samples. Table S3: Network topology indices corresponding to Figure 5. Figure S1: Locations of research sites. (a) Inner Mongolia map. (b) Sampling site locations. Figure S2: Rarefaction curves of (a) Simpson index and (b) Shannon index at ASV level for 8 halophytes.

## References

[1] Liu L., Gou X., Wang X., Yang M., Qie L., Pang G., Wei S., Zhang F., Li Y., Wang Q. (2024). Relationship between extreme climate and vegetation in arid and semi-arid mountains in China: A case study of the Qilian Mountains. Agric. For. Meteorol..

[2] Dong L., Li M.X., Li S., Yue L.X., Ali M., Han J.R., Lian W.H., Hu C.J., Lin Z.L., Shi G.Y. (2024). Aridity drives the variability of desert soil microbiomes across north-western China. Sci. Total Environ..

[3] Zhang S., Chen Y., Zhou X., Zhang Y. (2024). Climate and human impact together drive changes in ecosystem multifunctionality in the drylands of China. Appl. Soil Ecol..

[4] Rahman M.M., Mostofa M.G., Keya S.S., Siddiqui M.N., Ansary M.M.U., Das A.K., Rahman M.A., Tran L.S. (2021). Adaptive Mechanisms of Halophytes and Their Potential in Improving Salinity Tolerance in Plants. Int. J. Mol. Sci..

[5] Flowers T.J., Colmer T.D. (2008). Salinity tolerance in halophytes. New Phytol..

[6] Chen J., Wang Y. (2024). Understanding the salinity resilience and productivity of halophytes in saline environments. Plant Sci..

[7] Ji X., Xia Y., Zhang H., Cui J.L. (2022). The microscopic mechanism between endophytic fungi and host plants: From recognition to building stable mutually beneficial relationships. Microbiol. Res..

[8] Siddiqui Z.S., Wei X., Umar M., Abideen Z., Zulfiqar F., Chen J., Hanif A., Dawar S., Dias D.A., Yasmeen R. (2021). Scrutinizing the Application of Saline Endophyte to Enhance Salt Tolerance in Rice and Maize Plants. Front. Plant Sci..

[9] Morales-Vargas A.T., Lopez-Ramirez V., Alvarez-Mejia C., Vazquez-Martinez J. (2024). Endophytic Fungi for Crops Adaptation to Abiotic Stresses. Microorganisms.

[10] Ameen M., Mahmood A., Sahkoor A., Zia M.A., Ullah M.S. (2024). The role of endophytes to combat abiotic stress in plants. Plant Stress.

[11] Raza T., Qadir M.F., Khan K.S., Eash N.S., Yousuf M., Chatterjee S., Manzoor R., ru Rehman S., Oetting J.N. (2023). Unraveling the potential of microbes in decomposition of organic matter and release of carbon in the ecosystem. J. Environ. Manag..

[12] Gupta S., Schillaci M., Walker R., Smith P.M.C., Watt M., Roessner U. (2020). Alleviation of salinity stress in plants by endophytic plant-fungal symbiosis: Current knowledge, perspectives and future directions. Plant Soil.

[13] Alam B., Li J., Ge Q., Khan M.A., Gong J., Mehmood S., Yuan Y., Gong W. (2021). Endophytic Fungi: From Symbiosis to Secondary Metabolite Communications or Vice Versa?. Front. Plant Sci..

[14] Fontana D.C., de Paula S., Torres A.G., de Souza V.H.M., Pascholati S.F., Schmidt D., Dourado Neto D. (2021). Endophytic Fungi: Biological Control and Induced Resistance to Phytopathogens and Abiotic Stresses. Pathogens.

[15] Akram S., Ahmed A., He P., He P., Liu Y., Wu Y., Munir S., He Y. (2023). Uniting the Role of Endophytic Fungi against Plant Pathogens and Their Interaction. J. Fungi.

[16] Xu D., Yu X., Yang J., Zhao X., Bao Y. (2020). High-Throughput Sequencing Reveals the Diversity and Community Structure in Rhizosphere Soils of Three Endangered Plants in Western Ordos, China. Curr. Microbiol..

[17] Liu Z., Wang C., Yang X., Liu G., Cui Q., Indree T., Ye X., Huang Z. (2023). The Relationship and Influencing Factors between Endangered Plant Tetraena mongolica and Soil Microorganisms in West Ordos Desert Ecosystem, Northern China. Plants.

[18] Kefu Z., Hai F., Ungar I.A.J.P.S. (2002). Survey of halophyte species in China. Plant Sci..

[19] Xu D., Yu X., Chen J., Liu H., Zheng Y., Qu H., Bao Y. (2023). Arbuscular Mycorrhizae Fungi Diversity in the Root–Rhizosphere–Soil of Tetraena mongolica, Sarcozygium xanthoxylon, and Nitraria tangutorum Bobr in Western Ordos, China. Agronomy.

[20] Li E., Huang Y., Chen H., Zhang J. (2019). Floristic diversity analysis of the Ordos Plateau, a biodiversity hotspot in arid and semi-arid areas of China. Folia Geobot..

[21] Ma X., Chao L., Li J., Ding Z., Wang S., Li F., Bao Y. (2021). The Distribution and Turnover of Bacterial Communities in the Root Zone of Seven Stipa Species Across an Arid and Semi-arid Steppe. Front. Microbiol..

[22] Chen J., Zheng Y., Guo Y., Li F., Xu D., Chao L., Qu H., Wang B., Ma X., Wang S. (2021). Differences in microbial communities from Quaternary volcanic soils at different stages of development: Evidence from Late Pleistocene and Holocene volcanoes. Catena.

[23] Chen J., Xu D., Liu H., Chao L., Zheng Y., Qu H., Li F., Mo L., Wang B., Cheng B. (2021). Quaternary volcanic activities influence core soil microorganisms in a typical steppe. Catena.

[24] Zhang S., Ni X., Arif M., Yuan Z., Li L., Li C. (2020). Salinity influences Cd accumulation and distribution characteristics in two contrasting halophytes, *Suaeda glauca* and *Limonium aureum*. Ecotoxicol. Environ. Saf..

[25] Xu Z., Wang M., Ren T., Li K., Li Y., Marowa P., Zhang C. (2021). Comparative transcriptome analysis reveals the molecular mechanism of salt tolerance in *Apocynum venetum*. Plant Physiol. Biochem..

[26] Kerstiens G., Tych W., Robinson M.F., Mansfield T.A.J.N.P. (2010). Sodium-related partial stomatal closure and salt tolerance of *Aster tripolium*. New Phytol..

[27] Yuan F., Leng B., Wang B. (2016). Progress in Studying Salt Secretion from the Salt Glands in Recretohalophytes: How Do Plants Secrete Salt?. Front. Plant Sci..

[28] Ma X.z., Wang X.p. (2020). Aboveground and belowground biomass and its’ allometry for Salsola passerina shrub in degraded steppe desert in Northwestern China. Land. Degrad. Dev..

[29] Zuo Y., Li X., Yang J., Liu J., Zhao L., He X. (2021). Fungal Endophytic Community and Diversity Associated with Desert Shrubs Driven by Plant Identity and Organ Differentiation in Extremely Arid Desert Ecosystem. J. Fungi.

[30] Manici L.M., Caputo F., De Sabata D., Fornasier F. (2024). The enzyme patterns of Ascomycota and Basidiomycota fungi reveal their different functions in soil. Appl. Soil. Ecol..

[31] Li W., Li Z., Liu Y., Nie X., Zheng H., Zhang G., Wang S., Ma Y. (2023). Soil nutrients shape the composition and function of fungal communities in abandoned ancient rice terraces. J. Environ. Manag..

[32] Challacombe J.F., Hesse C.N., Bramer L.M., McCue L.A., Lipton M., Purvine S., Nicora C., Gallegos-Graves V., Porras-Alfaro A., Kuske C.R. (2019). Genomes and secretomes of Ascomycota fungi reveal diverse functions in plant biomass decomposition and pathogenesis. BMC Genom..

[33] Huang Q., Jiao F., Huang Y., Li N., Wang B., Gao H., An S. (2021). Response of soil fungal community composition and functions on the alteration of precipitation in the grassland of Loess Plateau. Sci. Total Environ..

[34] Chen L., Liang Y., Song T., Anjum K., Wang W., Yu S., Huang H., Lian X.Y., Zhang Z. (2015). Synthesis and bioactivity of tripolinolate A from *Tripolium vulgare* and its analogs. Bioorg Med. Chem. Lett..

[35] Manici L.M., Caputo F., Fornasier F., Paletto A., Ceotto E., De Meo I. (2024). Ascomycota and Basidiomycota fungal phyla as indicators of land use efficiency for soil organic carbon accrual with woody plantations. Ecol. Indic..

[36] Wu X., Hu H., Li S., Zhao J., Li J., Zhang G., Li G., Xiu W. (2022). Chemical fertilizer reduction with organic material amendments alters co-occurrence network patterns of bacterium-fungus-nematode communities under the wheat–maize rotation regime. Plant Soil.

[37] Wang Q., Xu J., Li D., Zhang J., Zhao B. (2024). Salinity-induced variations in wheat biomass are regulated by the Na(+):K(+) ratio, root exudates, and keystone species. Sci. Total Environ..

[38] Hyde K.D., Jones E.B.G., Liu J.-K., Ariyawansa H., Boehm E., Boonmee S., Braun U., Chomnunti P., Crous P.W., Dai D.-Q. (2013). Families of Dothideomycetes. Fungal Divers..

[39] Onlamun T., Boonthavee A., Brooks S. (2023). Diversity and Advantages of Culturable Endophytic Fungi from Tea (Camellia sinensis). J. Fungi.

[40] Chen K.H., Miadlikowska J., Molnar K., Arnold A.E., U’Ren J.M., Gaya E., Gueidan C., Lutzoni F. (2015). Phylogenetic analyses of eurotiomycetous endophytes reveal their close affinities to Chaetothyriales, Eurotiales, and a new order—Phaeomoniellales. Mol. Phylogenet. Evol..

[41] Recek N., Zhou R., Zhou R., Te’o V.S.J., Speight R.E., Mozetic M., Vesel A., Cvelbar U., Bazaka K., Ostrikov K.K. (2018). Improved fermentation efficiency of S. cerevisiae by changing glycolytic metabolic pathways with plasma agitation. Sci. Rep..

[42] Alberghina L., Mavelli G., Drovandi G., Palumbo P., Pessina S., Tripodi F., Coccetti P., Vanoni M. (2012). Cell growth and cell cycle in Saccharomyces cerevisiae: Basic regulatory design and protein-protein interaction network. Biotechnol. Adv..

[43] He C., Meng D., Li W., Li X., He X. (2023). Dynamics of Endophytic Fungal Communities Associated with Cultivated Medicinal Plants in Farmland Ecosystem. J. Fungi.

[44] Abrego N., Roslin T., Huotari T., Tack A.J.M., Lindahl B.D., Tikhonov G., Somervuo P., Schmidt N.M., Ovaskainen O. (2020). Accounting for environmental variation in co-occurrence modelling reveals the importance of positive interactions in root-associated fungal communities. Mol. Ecol..

[45] Tian L., Chen P., Gao Z., Gao X., Feng B. (2022). Deciphering the distinct mechanisms shaping the broomcorn millet rhizosphere bacterial and fungal communities in a typical agricultural ecosystem of Northern China. Plant Soil.

[46] Zhong F., Fan X., Ji W., Hai Z., Hu N., Li X., Liu G., Yu C., Chen Y., Lian B. (2022). Soil Fungal Community Composition and Diversity of Culturable Endophytic Fungi from Plant Roots in the Reclaimed Area of the Eastern Coast of China. J. Fungi.

[47] Li J., Xie T., Zhu H., Zhou J., Li C., Xiong W., Xu L., Wu Y., He Z., Li X. (2021). Alkaline phosphatase activity mediates soil organic phosphorus mineralization in a subalpine forest ecosystem. Geoderma.

[48] Kappaun K., Piovesan A.R., Carlini C.R., Ligabue-Braun R. (2018). Ureases: Historical aspects, catalytic, and non-catalytic properties—A review. J. Adv. Res..

[49] Zhou S., Chen L., Wang J., He L., Wang J., Ren C., Guo Y., Zhao F. (2022). Stronger microbial decay of recalcitrant carbon in tropical forests than in subtropical and temperate forest ecosystems in China. Catena.

[50] Sharma R.K., Cox M.S., Oglesby C., Dhillon J.S. (2024). Revisiting the role of sulfur in crop production: A narrative review. J. Agric. Food Res..

[51] Xiao W., Zhang Y., Chen X., Sha A., Xiong Z., Luo Y., Peng L., Zou L., Zhao C., Li Q. (2024). The Diversity and Community Composition of Three Plants’ Rhizosphere Fungi in Kaolin Mining Areas. J. Fungi.

[52] Xing J., Li X., Li Z., Wang X., Hou N., Li D. (2024). Remediation of soda-saline-alkali soil through soil amendments: Microbially mediated carbon and nitrogen cycles and remediation mechanisms. Sci. Total Environ..

[53] Suzuki T. (2021). The expanding world of tRNA modifications and their disease relevance. Nat. Rev. Mol. Cell Biol..

[54] Nobori T., Wang Y., Wu J., Stolze S.C., Tsuda Y., Finkemeier I., Nakagami H., Tsuda K. (2020). Multidimensional gene regulatory landscape of a bacterial pathogen in plants. Nat. Plants.

[55] Liers C., Bobeth C., Pecyna M., Ullrich R., Hofrichter M. (2010). DyP-like peroxidases of the jelly fungus Auricularia auricula-judae oxidize nonphenolic lignin model compounds and high-redox potential dyes. Appl. Microbiol. Biotechnol..

[56] Allmér J., Stenlid J., Dahlberg A. (2009). Logging-residue extraction does not reduce the diversity of litter-layer saprotrophic fungi in three Swedish coniferous stands after 25 years. Can. J. For. Res..

