# Supplementary material for: Diversity and Functional Insights into Endophytic Fungi in Halophytes from West Ordos Desert Ecosystems
Source: J Fungi (Basel). 2025 Jan 4;11(1):30. doi: 10.3390/jof11010030 (PMC11766765; doi:10.3390/jof11010030)
Supplement: Supplementary file 1 [file jof-11-00030-s001.zip › jof-3345074-supplementary.pdf]

SUPPLEMENTARY INFORMATION

Diversity and Functional Insights into Endophytic Fungi in Halophytes from West Ordos Desert Ecosystems

Xingzhe Wang[a,b], Yan Zhang[a,b], Jingpeng Li[a,b], Yiteng Ding[a,b], Xiaodan Ma[a,b], Peng Zhang[a,b], Haijing Liu[a,b] , Jie Wei[a,b], Yuying Bao[a, b] *

[a] *Key Laboratory of Forage and Endemic Crop Biotechnology, Ministry of Education, School of Life Sciences, Inner Mongolia University, Hohhot, 010010, PR China*

[b] *State Key Laboratory of Reproductive Regulatory and Breeding of Grassland Livestock, Inner Mongolia University, Hohhot, 010010, PR China*

*Corresponding author Address: Inner Mongolia Univ, 235 West Univ Rd, Hohhot 010021, Inner Mongolia, Peoples R China

*Corresponding author. E-mail address: ndbyy@imu.edu.cn

TABLES

Table S1 Basic spatial geographic parameters information of the study site.

Sites	Site code	Sampled Plants	Location	Altitude (m)	pH	SM (%)	Sampling time
Ordos City	S1	*Reaumuria kaschgarica* (Rk)<br>*Nitraria tangutorum* (Nt)	E106°50′11.57″<br>N39°51′25.37″	1178	8.43	0.85	2021.8.16
Wuhai City	S2	*Limonium aureum* (La)<br>*Tripolium vulgare* (Tv)	E106°53′7.08″<br>N39°44′6.64″	1224	8.10	0.68	2021.8.16
Alxa City	S3	*Suaeda glauca* (Sg)	E106°45′18.85″<br>N39°41′45.33″	1069	7.99	2.23	2021.8.17
Wuhai City	S4	*Apocynum venetum* (Av)	E106°47′24.96″<br>N39°40′36.00″	1066	7.81	0.89	2021.8.17
Wuhai City	S5	*Reaumuria kaschgarica* (Rk)	E106°49′27.08″<br>N39°36′49.04″	1254	7.93	1.15	2021.8.18
Wuhai City	S6	*Reaumuria trigyna* (Rt)<br>*Salsola passerina* (Sp)	E106°52′34.32″<br>N39°32′6.96″	1351	8.31	0.86	2021.8.18

Table S2 Alpha-diversity indices of six samples

Sample	Chao1	Goods Coverage	Shannon	Simpson	Observed specie	Pielou's evenness
Reaumuria trigyna (Rt)	75.241	0.999975	4.040	0.914983	74.80	0.650
Reaumuria trigyna (Rt)	154.051	0.999974	4.699	0.895719	153.60	0.647
Limonium aureum (La)	148.210	0.999947	3.951	0.875747	147.13	0.5493
Salsola passerina (Sp)	80.310	0.999974	4.170	0.911612	79.80	0.661
Nitraria tangutorum (Nt)	33.423	0.999986	0.274	0.051549	33.23	0.054
Suaeda glauca (Sg)	28.571	0.999983	1.369	0.376327	28.17	0.285
Apocynum venetum (Av)	693.673	0.999495	2.717	0.622756	684.53	0.290
Tripolium vulgare (Tv)	30.514	0.999983	1.750	0.552132	30.17	0.357

Table S3 Network topology indices corresponding to Figure 5

	Empirical Network Indexes								Random networks		
taxa	Number of vertice	Number of edge	Density	Degree centralization	Transitivity	Average nearest neighbor degree	Average path lenghth	Modularity	Average nearest neighbor degree	Average path lenghth	Modularity
**Recretohalophytes**	65	197	0.10	766	0.60	7.23	3.62	0.61	6.94	2.48	0.33
**Euhalophytes**	51	135	0.11	444	0.72	6.42	4.10	0.53	6.17	2.51	0.35
**Pseudohalophytes**	29	41	0.11	121	0.57	3.42	2.29	0.58	3.66	3.12	0.47

FIGURE

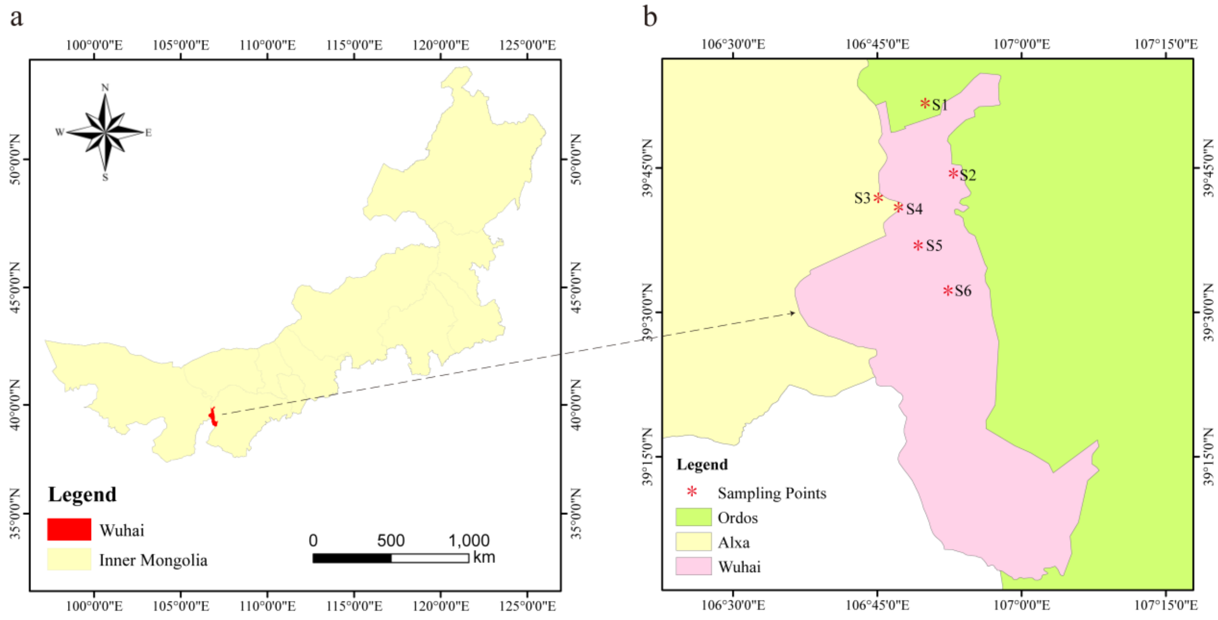

Figure S1 Locations of the research sites. (a) Inner Mongolia map. (b) Sampling site locations.

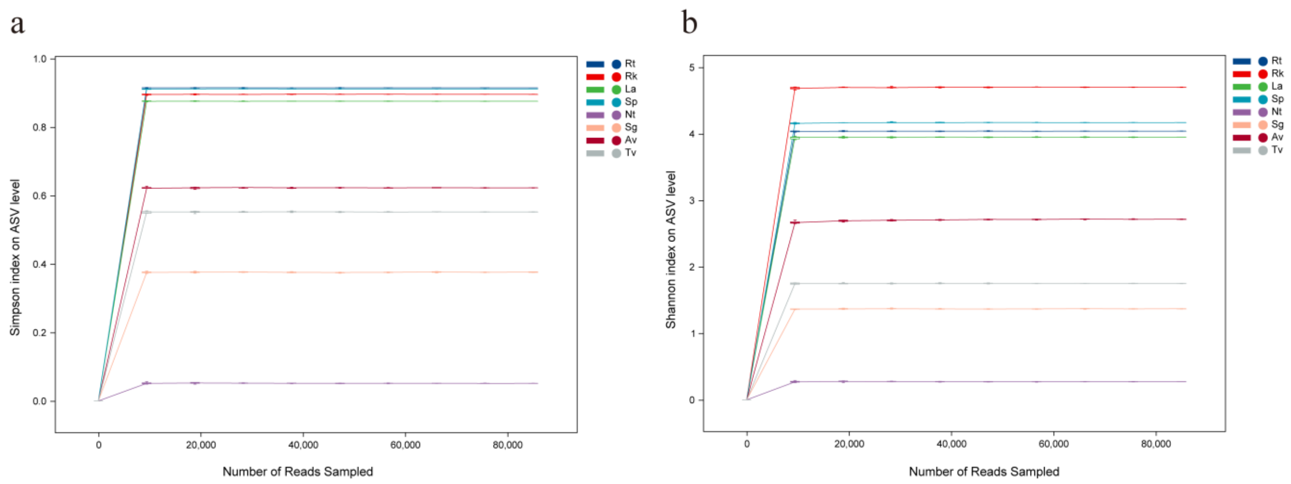

Figure S2 The rarefaction curves of (a) Simpson index and (b) Shannon index at ASV level for 8 halophytes.
